# Approaching community priorities in youth sports injury prevention research

**DOI:** 10.1186/s40621-020-00261-2

**Published:** 2020-07-06

**Authors:** Zachary Y. Kerr, Paula Gildner, Aliza K. Nedimyer, Avinash Chandran, Melissa C. Kay, K. Hunter Byrd, Johna K. Register-Mihalik

**Affiliations:** 1 Department of Exercise and Sport Science, Injury Prevention Research Center, University of North Carolina at Chapel Hill, 313 Woollen Gym CB#8700, Chapel Hill, NC 27599-8700 USA; 2 Injury Prevention Research Center, University of North Carolina at Chapel Hill, Sheryl-Mar South Building, 521 S Greensboro St, Carrboro, NC 27510 USA; 3 Human Movement Science Curriculum, University of North Carolina at Chapel Hill, 209 Fetzer Hall, CB#8700, Chapel Hill, NC 27599-8700 USA; 4 Datalys Center for Sports Injury Research and Prevention, 401 West Michigan Street, Suite 500, Indianapolis, IN 46202 USA; 5 School of Health Professions, University of Southern Mississippi, Harkins Hall (EHH), Hattiesburg, MS 224 USA; 6 Department of Exercise and Sport Science, Injury Prevention Research Center, University of North Carolina, 125 Fetzer Hall CB#8700, Chapel Hill, NC 27599-8700 USA

**Keywords:** Traumatic brain injury, Youth sports, Emergency preparedness, Community priorities, Stakeholders

## Abstract

**Background:**

Research in youth sports is often complex. As interest in youth sports injury prevention grows, scientists should consider community priorities beyond a specific research study.

**Main text:**

This commentary discusses the authors’ personal experiences researching concussion prevention in middle school sports, as the overarching community faced multiple challenges. These challenges included a series of weather-related emergencies that resulted in a shift in the community’s priorities, multi-day school closures, and cancellations of sports activities and meetings. We discuss the importance of considering community priorities and providing support as scientists, colleagues, and members of the communities in which we conduct research.

**Conclusion:**

Scientists should consider the changing circumstances and dynamics surrounding community priorities in order to help drive their research-based decisions and ensure successful and respectful applications of research based on community values and priorities.

## Introduction

Research in youth sports is often complex. First, youth are a “vulnerable” population requiring additional protections (Belmont Report 1979; U.S. Department of Health and Human Services 2016). Further, working with youth involves building trust with them, their legal guardians, and the persons/organizations with whom youth are associated (Kerr et al. 2018).

Second, alongside surveillance efforts within middle school, high school, and recreational league/club sport settings, (Pierpoint et al. 2018; Kerr et al. 2016; Dompier et al. 2015; Kerr et al. 2017) interest in applied field research is growing. With such research, scientists may lack a degree of experimental oversight (e.g., inability to control for all extraneous variables, consistency in exposure to intervention across/within sites). Still, applied field research can examine phenomenon in “real world” settings with opportunities for participants to directly participate in intervention development (Ahmed et al. 2018).

Finally, scientists may likely conduct research locally within their own communities. They may be linked to the targeted youth population as parents or coaches. This ethical dilemma, described as “divided loyalties” by Bell and Nutt, (Bell and Nutt 2002) can consequently manifest negatively if scientists do not consider how to best approach such community-based research.

Scientists must carefully consider the communities in which they work and identify the dynamics of these communities’ priorities. These priorities likely vary by community, setting, and research topic, but in youth sports may include safety, competition, skills development, and personal development (Kerr et al. 2018). Although it can be argued that scientists are trained to innately be cognizant of culture and norms that may influence community priorities, it is nevertheless important to have these discussions, particularly while training the next generation of scientists.

### Considering these issues in our middle school concussion prevention research

We found that such discussions of the priorities of the community have been important in our ongoing research that examines middle school sport concussion prevention. The study, approved by the Institutional Review Board at the University of North Carolina at Chapel Hill, aimed to develop and implement an educational intervention to improve concussion management in middle school sports (Kerr et al. 2018). Using a sample of local middle schools, we conducted formative ethnographic research and interviews with the middle school sport stakeholders (e.g., parents, coaches, athletes, staff, administrators), and provided concussion education and prevention materials to the middle schools. Such an approach to interact with stakeholders prior to the development and dissemination of a concussion intervention had been previously utilized in other settings (Craig et al. 2019). However, the middle school sports setting was distinct from other settings and needed a concussion education and prevention framework that could suit its unique needs (Kerr et al. 2018). Compared to high schools, middle schools may be less likely to have an on-site athletic trainer or enact concussion-related policy (Kerr et al. 2018; DePadilla et al. 2020). As in other emerging and under-studied settings, (Caine and Provance 2018; Emery 2018) formative research in this area was needed to identify community priorities, as related to the specific sports settings and overall within the middle school. We hoped that doing so would help to recognize the requisite factors to aid the successful implementation of sport-related concussion education and prevention strategies.

During the study, the community faced multiple challenges, resulting in multi-day school closures and the cancellations of sports activities and meetings. Weather-related emergencies, such as hurricanes and winter storms, caused outdoor fields to be unusable and directly impacted sport-related events. Additional atypical events (e.g., water main break) also caused further school closures.

In the midst of these challenges, our research and interaction with middle school sport stakeholders came to a halt due to changing priorities. A number of community members were personally affected (e.g., major damage to their homes or homes of loved ones, resultant financial burden). Consequently, some stakeholders noting that middle school sports and our concurrent research and prevention efforts were understandably not a priority at the time. Members of our research staff also had to contend with similar tangible damage; one staff member’s family home was flooded and another staff member had a tree fall through the roof of her home. We were aware of the need to proceed in a manner that was respectful and needed to reflect on our next steps in how best to respect our stakeholders’ priorities.

We knew that continuing to conduct research without acknowledging the challenges in our community would not be responsible. Further, these affected individuals were members from our own community and we felt our next actions could either support or inhibit cultivating a sense of trust. There were members of the community that were still interested in the research and wanted to continue the process over the long-term, but understandably had other immediate priorities. The steps we took in response considered feedback and input from all levels of the study, including community members, study staff, and our funding agency.

Although we had some ideas of what steps could be taken to provide support (e.g., contacting and checking in with our schools, continuing to attend practices and games when possible even if to simply cheer on the athletes), we also had to be responsive to the circumstances as they changed and evolved. We wanted to ensure that we were respectful and were not perceived as solely caring about our own research endeavors. For those particularly affected, we aimed to provide space to allow them to assess their options in terms of disaster-related outcomes, such as damage to their and their loved ones’ property and mental health. This also extended to our own staff - work schedules were modified, with substitutes completing tasks as needed. In collaboration with our research community, we worked to modify data collection procedures and efforts so that they fit within the transition periods needed as everyone returned to a sense of normalcy. In some cases, this included not collecting data at all and rather, maintaining communication with coaches and administrators. We rescheduled meetings and were flexible as school personnel worked with parents and athletes to resume sport activity. Throughout this period, we kept our funding agency abreast of the evolving circumstances and any potential changes/deviations from the proposed protocol (e.g., shorter fall and winter sport season lengths than previously anticipated). We appreciate that the funding agency was supportive of such communication.

## Conclusions

Although the challenges that the community in our research study encountered were likely uncommon, discussions of community priorities are salient across all research disciplines and among all members of the research community, from the most senior research team leader to undergraduate students in a research methodology course. This discussion may be most imperative in the context of the COVID-19 pandemic. The research community has ceased all face-to-face research and continues to debate how to proceed with future research efforts.

Scientists’ reactions to communities’ changing priorities can impact the development and maintenance of trust between science and the public. Further, applied field research can require scientists to respond to conditions that fall outside of the planned study timeline or the entire scope of the study. Such response should emphasize flexibility to the community’s changing priorities. Such changes may cause scientists to become concerned about their ability to complete objectives and milestones set forth by their funding agencies. We recommend initiating and maintaining contact with one’s funding agency when such concerns arise, and proposing additional measures to achieve goals despite any setbacks. Further, it may be possible that large-scale issues may benefit from guidance from one’s Institutional Review Board. Research communities should consider their approaches when working with their populations of interest to help ensure successful and respectful practice of public health and injury prevention.

## Data Availability

Data sharing not applicable to this article as no datasets were generated or analyzed during the current study.
